# Supplementary material for: Autism-associated CHD8 keeps proliferation of human neural progenitors in check by lengthening the G1 phase of the cell cycle
Source: Biol Open. 2022 Sep 27;11(9):bio058941. doi: 10.1242/bio.058941 (PMC9548376; doi:10.1242/bio.058941)
Supplement: Supplementary information [file biolopen-11-058941-s1.pdf]

Figure S1 Coakley-Youngs et al.,

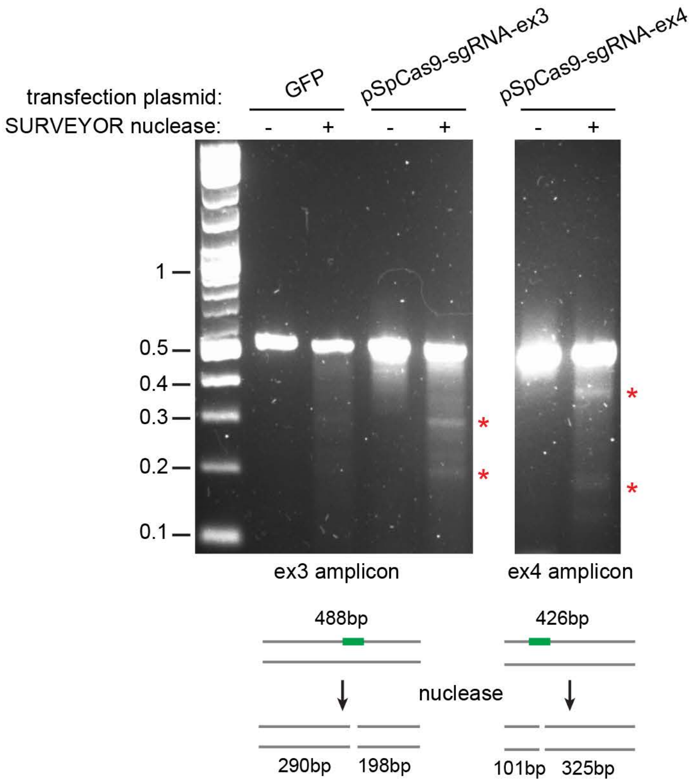

Fig. S1. Surveyor assay and TIDE results. (a) Surveyor assay performed on hESCs transfected with pSpCas9-ex3-gRNA and pSpCas9-ex4-gRNA. The cleavage pattern observed for both gRNAs (red asterisks) is consistent with a double strand break at the predicted site. The predicted positions of the DSB sites in these genomic amplicons are depicted below. These cleavage products are not observed in hESCs transfected with GFP.

Biology Open • Supplementary information

Figure S2 Coakley-Youngs et al.,

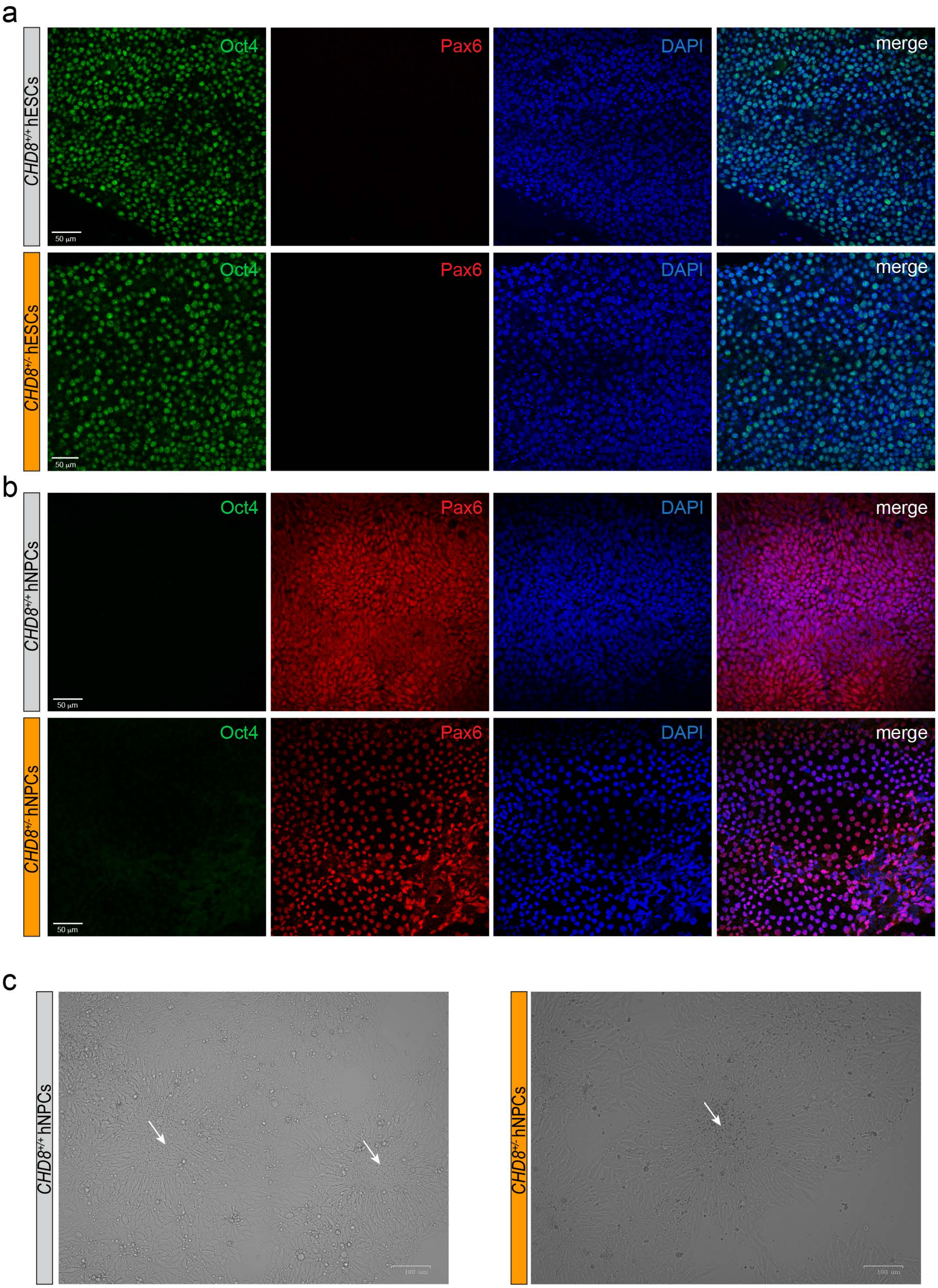

Fig. S2. Differentiation of *CHD8*$^{+/+}$ and *CHD8*$^{+/-}$ hESCs into NPCs. 3-color confocal imaging of Oct4, Pax6 and DAPI in wt and mutant hESCs (a) and NPCs (b). The vast majority of non-mitotic hESC cells (with intact nuclear envelope) are positive for Oct4, irrespective of genotype, and do not express Pax6 (a). Conversely, hESCs neuralized by dual SMAD inhibition are Pax6-positive and Oct4-negative (b). Occasionally (< 5% of cells), a DAPI-positive cell is not stained by either Oct4 or Pax6 in wt or mutant NPCs. (c) bright field images of neural rosettes in wt and mutant NPCs after 4 weeks of neural induction/differentiation.

Figure S3 Coakley-Youngs et al.,

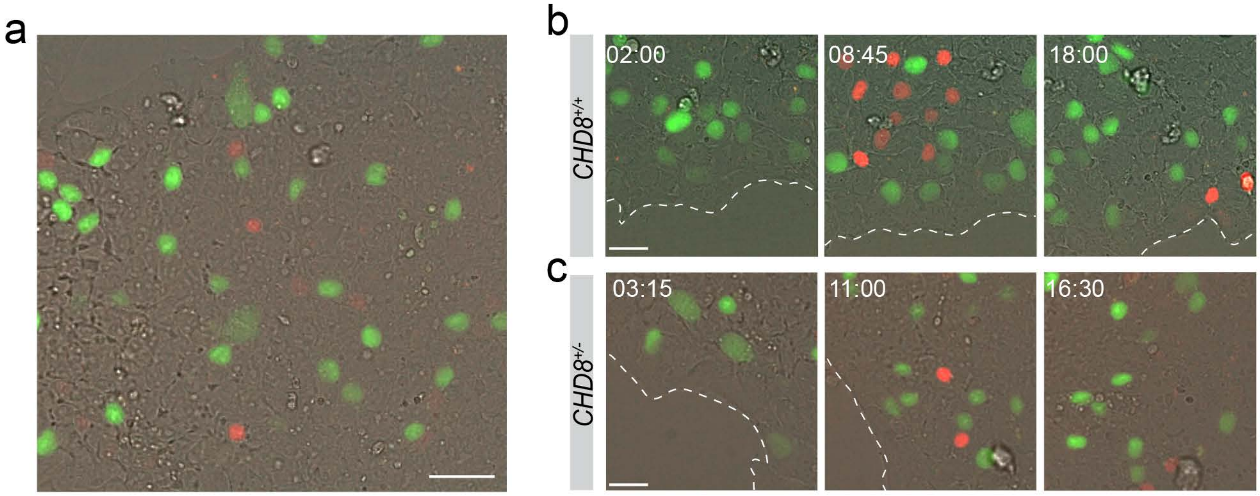

Fig. S3. (a) Image of a hESC colony transduced with FUCCI. The green and red channels are overlaid on top of a brightfield image. (b,c) Snapshots of FUCCI-transduced *CHD8*$^{+/+}$ and *CHD8*$^{+/-}$ hESCs taken over the course of ~ 20hrs. The white dotted line shows expansion of the stem cell colony over time. Time is indicated in hours and mins. Scale bar: 20 μm.

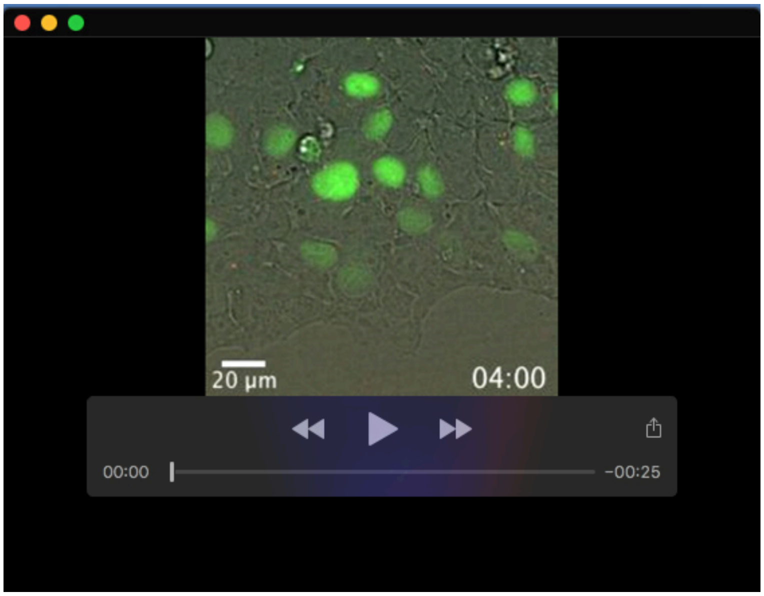

Movie 1. time-lapse imaging of FUCCI-expressing *CHD8*$^{+/+}$ hESCs (10 fps), shown in Figure 3d.

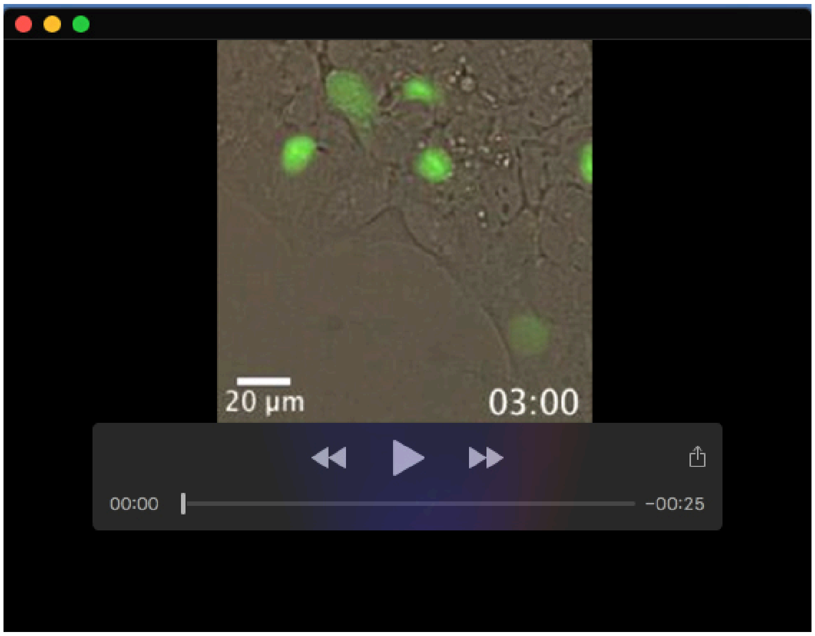

Movie 2. time-lapse imaging of FUCCI-expressing *CHD8*$^{+/-}$ hESCs (10 fps), shown in Figure 3e.

Biology Open • Supplementary information

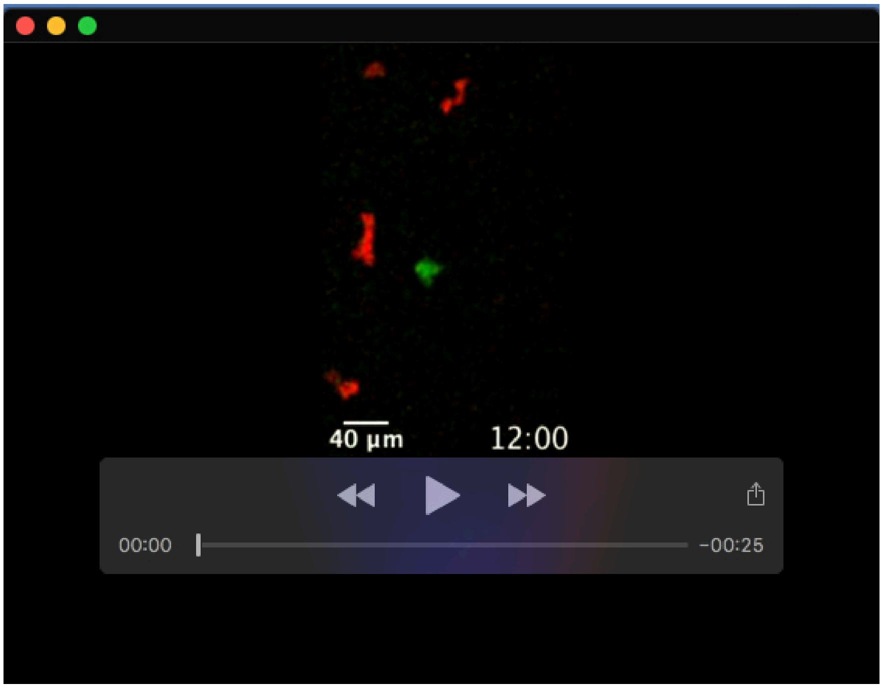

Movie 3. time-lapse imaging of FUCCI-expressing *CHD8*$^{+/+}$ hNPCs (10 fps), shown in Figure 4a.

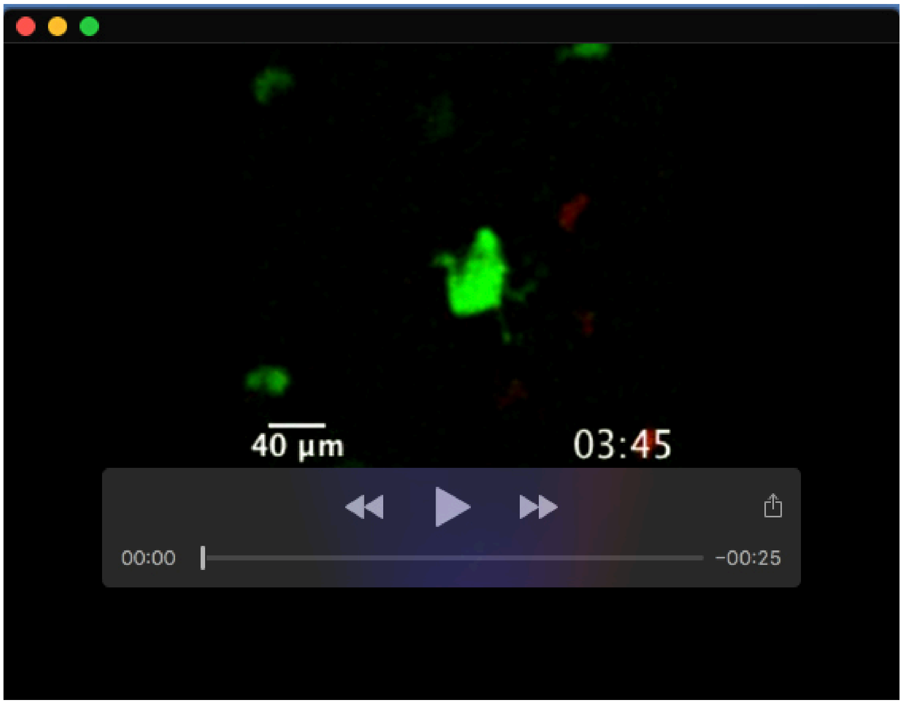

Movie 4. time-lapse imaging of FUCCI-expressing *CHD8*$^{+/-}$ hNPCs (10 fps), shown in Figure 4b.

Biology Open • Supplementary information

Matlab script

```
% This script uses a point-and-click strategy to segment and track FUCCI-labeled cells
% in a time series.
% The script displays the read and green FUCCI intensities (C1Int and C2Int) for all
% cells identified in a lineage.
% The scripts saves a spreadsheet containing coordinates of each click and
% corresponding FUCCI intensities in both channels.
% last modified by Marc Fivaz, October 2020.

clear all, close all
```

load up stacks

```
C1 = 'R-G07.tif'; % change this to the name of your red stack
InfoImage=imfinfo(C1);
mImage=InfoImage(1).Width
nImage=InfoImage(1).Height
NumberImages=length(InfoImage)

FinalImage=zeros(nImage,mImage,NumberImages,'uint8');
for i=1:NumberImages
  C1s(:,:,i)=imread(C1,'Index',i);
end

C2 = 'G-G07.tif'; %change this to the name of your green stack
InfoImage=imfinfo(C2);
mImage=InfoImage(1).Width;
nImage=InfoImage(1).Height
NumberImages=length(InfoImage)

FinalImage=zeros(nImage,mImage,NumberImages,'uint8');
for i=1:NumberImages
  C2s(:,:,i)=imread(C2,'Index',i);
end
```

local background subtraction

```
se = strel('disk', 30);
for k = 1:NumberImages
          C1s_bg(:,:,k) = imopen(C1s(:,:,k), se);
          C1s_corr(:,:,k) = C1s(:,:,k) - C1s_bg(:,:,k);
          C2s_bg(:,:,k) = imopen(C2s(:,:,k), se);
          C2s_corr(:,:,k) = C2s(:,:,k) - C2s_bg(:,:,k);
end
```

variables

Biology Open • Supplementary information

```
filename = 'G07_s2';
timeInterval = 915.86; % extract from meta data
FrameNumber = NumberImages;
time = [0:15.264:(NumberImages-1)*15.264];
time = time';
```

binary image from each channel

```
for i = 1:NumberImages
        TC1 = adaptthresh(C1s_corr(:,:,i), 0.15); % adaptive thresholding
        TC2 = adaptthresh(C2s_corr(:,:,i), 0.075); % adaptive thresholding
        bwC1(:,:,i) = imbinarize(C1s_corr(:,:,i), TC1);
        bwC2(:,:,i) = imbinarize(C2s_corr(:,:,i), TC2);
        bwC1(:,:,i) = imfill(bwC1(:,:,i), 'holes');
        bwC2(:,:,i) = imfill(bwC2(:,:,i), 'holes');
        bwC1(:,:,i) = bwareaopen(bwC1(:,:,i), 50); % minimum size
        bwC2(:,:,i) = bwareaopen(bwC2(:,:,i), 50); % minimum size
end
```

sum C1 and C2 binary stacks and visualize segmentation

```
for i = 1:NumberImages
        bw_sum(:,:,i) = bwC1(:,:,i) + bwC2(:,:,i);
        bw_sum_perim(:,:,i) = bwperim(bw_sum(:,:,i));
        ImMerge = imfuse(C1s_corr(:,:,i), C2s_corr(:,:,i), 'ColorChannels', [1 2 0]);
        ImOv(:,:,:,i) = imoverlay(ImMerge, bw_sum_perim(:,:,i), [1 1 1]);
end
```

interactive segmentation of cells and their progeny

```
for i = 1:NumberImages
        C1s_corr_adj(:,:,i) = imadjust(C1s_corr(:,:,i), [0.005 0.02], []);
        C2s_corr_adj(:,:,i) = imadjust(C2s_corr(:,:,i), [0 0.07], []);
        ImMerge = imfuse(C1s_corr_adj(:,:,i), C2s_corr_adj(:,:,i), 'ColorChannels', [1 2 0]);
        imshow(ImMerge, []), tqmax;
        C11s = C1s_corr(:,:,i);
        C22s = C2s_corr(:,:,i);
        [col,row] = ginput;

        for k = 1:length(col)
        bw2 = bwselect(bw_sum(:,:,i), col(k), row(k), 8);
        imshow(bw2);
        pause(0.05)
        CC = bwconncomp(bw2, 8);
        TF = isempty(CC.PixelIdxList);
        if TF == 1
```

Biology Open • Supplementary information

```
            C1Int(i,k) = mean(mean(C1s_bg(:,:,i)));
            C2Int(i,k) = mean(mean(C2s_bg(:,:,i)));
            elseif TF == 0
            C1Int(i,k) = mean(C11s(CC.PixelIdxList{1,1}));
            C2Int(i,k) = mean(C22s(CC.PixelIdxList{1,1}));
            end
            RInt(i,k) = C1Int(i,k);
            GInt(i,k) = C2Int(i,k);
            end
            allcoord2(:, 1) = col;
            allcoord2(:, 2) = row;
            allcoord2(:, 3) = 1:length(col);
            allcoord2(:, 4) = i;
            allcoord2(:,5) = C1Int(i,:)';
            allcoord2(:,6) = C2Int(i,:)';
            if i == 1
            allcoord = allcoord2;
            else
            allcoord = vertcat(allcoord, allcoord2);
            end
            clear allcoord2;
            clear C1Int;
            clear C2Int;
end
```

Plot intensity traces

```
figure, hold on
plot(time, GInt(:,1), 'g');
plot(time, RInt(:,1), 'r');
hold off
xlabel('time min');
ylabel('Int');
legend('S/G2/M', 'G1');
title('cell 1')

figure, hold on
plot(time, GInt(:,2), 'g');
plot(time, RInt(:,2), 'r');
hold off
xlabel('time min');
ylabel('Int');
legend('S/G2/M', 'G1');
title('cell 2');
```

Biology Open • Supplementary information

```
% repeat if lineages includes more than two daughter cells
```

create and export dataset

```
T = table(allcoord (:, 1), allcoord (:, 2), allcoord (:, 3), allcoord(:, 4), allcoord(:, 5), allcoord(:, 6));
T.Properties.VariableNames = {'column' 'row' 'cellnumber' 'frame' 'C1Int' 'C2Int'};
fname = strcat(filename, '_CoordInt.xlsx');
writetable(T, fname);
```

Biology Open • Supplementary information
